# Ethnic Differences in the Use of Complementary and Alternative Therapies Among Adults With Osteoarthritis

**Published:** 2006-06-15

**Authors:** Carla J Herman, Jenny M Dente, Peg Allen, William C Hunt

**Affiliations:** University of New Mexico Center on Aging. Dr Herman is also affiliated with Division of Geriatrics, Department of Internal Medicine, University of New Mexico School of Medicine; University of New Mexico School of Medicine, Albuquerque, NM. Dr Dente is now affiliated with Oregon Health and Sciences University, Portland, Ore; Division of Geriatrics, Department of Internal Medicine, University of New Mexico School of Medicine, Albuquerque, NM; Epidemiology and Cancer Control, University of New Mexico Health Sciences Center, Albuquerque, NM

## Abstract

**Introduction:**

The use of complementary and alternative medicine (CAM) in the United States has been rising steadily, especially among people with chronic conditions such as osteoarthritis. It has been suggested that ethnicity and acculturation may influence use of CAM. The purpose of this study was to assess the influence of ethnicity and acculturation on patterns of CAM use among Hispanic and non-Hispanic white adults with osteoarthritis.

**Methods:**

We conducted interviews in person, in English or Spanish, using a 255-item survey. We randomly selected participants aged 18 to 84 years from patients at university-based primary care outpatient clinics who had been diagnosed with osteoarthritis during the previous year. Measures included prevalence and types of CAM use, sociodemographic factors, self-reported ethnicity, and degree of acculturation according to language use.

**Results:**

The Hispanic (n = 218) and non-Hispanic white (n = 204) populations showed similar rates of overall current CAM use (65.5% Hispanic vs 67.8% NHW) at time of interview. However, although more Hispanics used oral herbs (*P* = .03) and magnets or copper jewelry (*P* = .03), more non-Hispanic whites used nutritional supplements (*P* < .001). Hispanics speaking primarily English mirrored patterns of CAM use among non-Hispanic whites. These effects persisted after controlling for age, sex, income, education, degree of disability, and disease duration.

**Conclusion:**

In this population, ethnicity was a significant influence on patterns of CAM use but did not affect overall rates of use. Some differences were more pronounced among Spanish-speaking Hispanics, reflecting the incorporation of folk or traditional remedies into their health care practices.

## Introduction

Complementary and alternative medicine (CAM) describes a broad category of health care practices that are not currently a part of conventional Western medicine (1). From 1990 to 1997, the number of individuals reporting the use of CAM within the previous year increased from 34% to 42% (2), and a recent national study found that 62% of telephone survey respondents had used some CAM therapy within 12 months of the survey (3). Several explanations have been proposed for the apparent surge in CAM popularity, including dissatisfaction with our current health care system, a failure of conventional treatments or an attempt to avoid drug side effects, a desire for more personal control over health care decisions, the influence of our society's recent focus on health promotion and disease prevention, and the impact of family or cultural background (2-7).

Although some studies of primary care patients have charted increased CAM use among young, white, well-educated, and more economically secure populations (2-4,8-10), other research has pointed out that CAM use is also high among low-income or rural groups, highlighting again the variety of reasons for which individuals may use CAM (2,3,5,11). Wootton and Sparber suggested a bimodal distribution of CAM use, in which higher-income groups use disposable income for CAM products and services that supplement their standard health care and ethnic minority and lower-income groups use traditional healing as a substitute for conventional care (11). The evolution of medical practices among ethnic minorities is seen as part of the larger process of socialization within U.S. culture; the socialization process has also been labeled *acculturation* as the values, behaviors, and norms of individuals within ethnic minorities are gradually modified through exposure to a new culture. Degree of acculturation may potentially influence patterns of medical care (12). Folk remedies, defined loosely as layperson's medicine, seem to be used largely in ethnic minority populations, with frequency of use related inversely to level of acculturation (4,12). Among ethnic minority populations, Hispanics appear to use traditional or folk remedies — most commonly home or self-care practices — significantly more often than other minority groups (4,12). Still, in light of the scarcity of research on patterns of CAM use among ethnic minorities, it has been difficult to determine trends and CAM modalities specific to Hispanic populations.

It has been suggested that chronic musculoskeletal conditions provide an ideal framework in which to research CAM use because they are prevalent, have no known cure, are characterized by chronic pain, and often adversely affect normal function (13); they are also among the most frequently cited reasons for using CAM (2-4,7,8,14,15). Osteoarthritis  is the most prevalent of these conditions (16). Few studies, however, have surveyed patterns of CAM use among the osteoarthritis population, and fewer still have investigated variations of CAM use among different groups within the osteoarthritis population (8,15,17-20).

This study documented patterns of use of CAM therapies among adults with osteoarthritis in a New Mexico primary care clinic population to assess whether there were significant differences in CAM use between Hispanics and non-Hispanic whites (NHWs) and whether ethnic variations were influenced by level of acculturation, socioeconomic status, and education.

## Methods

### Sample selection and recruitment 

The individuals included in this study were part of a larger study of CAM use (21). The population included all patients aged 18 to 84 years who visited one of six primary care clinics of the University of New Mexico Hospital system in Albuquerque, NM, from June 2000 through May 2001 and were diagnosed with osteoarthritis, rheumatoid arthritis, or fibromyalgia. We randomly selected participants within categories of diagnosis, sex, and ethnic group (Hispanic and NHW) from the hospital outpatient management database. To obtain more Hispanics, more men, and more rheumatoid arthritis participants, patients in these categories were sampled at higher rates than NHW women with diagnoses of osteoarthritis or fibromyalgia. The sampling strata were based on the clinic-assigned ethnicity and diagnostic group, although self-reported ethnicity is used in the analysis.

Of 1210 eligible patients, 612 (50.6%) participated in the larger study (21). Study participants and nonparticipants were similar in sex and age but differed in ethnicity and clinic diagnosis. The response rate among participants with clinic-designated Hispanic ethnicity (45%) was significantly lower than among NHWs (55%) (*P* < .001), and patients with rheumatoid arthritis were significantly more likely to participate than patients with osteoarthritis (*P* = .006). Among the 612 participants, 42% had a clinic designation of Hispanic ethnicity, which was significantly lower than the 48% self-reported prevalence of Hispanic ethnicity (*P* < .001 by McNemar's test). The crude agreement between clinic and self-reported ethnicity was 89.9% with a κ value of 79.6. For this paper, patients with clinic diagnoses of rheumatoid arthritis (n = 95) and self-reported fibromyalgia (n = 95) were excluded, and the analysis was limited to those with osteoarthritis (n = 422).

After patients were selected from the clinic database, we obtained active written consent to contact them from their primary-care provider and then mailed them an invitation to complete an in-person interview. Trained interviewers followed up the introductory letters with telephone calls in English or Spanish, inviting individuals to participate and screening the eligibility of those interested; to be eligible, participants had to self-identify as Hispanic or NHW and speak either English or Spanish. Potential participants were informed that the interview would ask about ways they managed their arthritis on their own, beyond what their primary care provider prescribed and recommended; the explanation did not use CAM terminology. The interview took an average of 45 minutes to complete. Eligible individuals signed an informed consent form just before their interview. This study received approval from the institutional review board at the University of New Mexico Health Sciences Center.

### Survey instrument 

We developed the survey instrument after an extensive literature review, a review of previous surveys of CAM use, consultation with Centers for Disease Control and Prevention (CDC) staff, and focus group interviews. We designed the survey to elicit information on CAM use for arthritis only. We used the Behavioral Risk Factor Surveillance System and Quality of Life questionnaires (22,23) to assess demographic information, self-reported type of arthritis, perceived health status, and comorbidities. We used the Stanford Health Assessment Questionnaire (HAQ) (24) to measure functional ability, the five-item Arthritis Helplessness Index (25) to address perceived ability to manage arthritis (25), a four-item medical skepticism scale (26) to assess attitudes toward conventional medical therapy, and the Wong-Baker Faces Pain Scale to assess pain (27). We also included scales evaluating fatigue and sleep problems (28,29). Finally, to measure acculturation, we used a five-item scale to evaluate the extent to which Spanish and English were used in day-to-day life. The scale was extrapolated from a 12-item scale assessing language use, media preferences, and ethnic social relations (30). Marin et al found that the five-item language scale correlated strongly with the Mexican American and Central American respondents' generation in the United States, length of U.S. residence, age at arrival in the United States, ethnic self-identification, and an independent acculturation index (30). The use of prayer as a CAM strategy to manage osteoarthritis, although asked in the questionnaire, was excluded from the analyses because subjects were unable to distinguish between prayer for overall health and for osteoarthritis management.

Prevalidated Spanish translations were used for the HAQ (31); demographic, health status, and comorbidity items (22); Arthritis Helplessness Index (32); and acculturation measures (30). The remainder of the survey was translated by a Mexican-born translator, edited by two bilingual interviewers to ensure comprehension by non-Mexican Spanish-speakers, and back-translated to English by a bilingual health researcher to ensure equivalent meaning. We piloted the Spanish version among several adults with arthritis who were known to the researchers and who were not in the study sample and then made minor revisions.

### Data collection

During 2001 and 2002, trained interviewers conducted in-person interviews in English or Spanish in a private room either in the participant's home or in one of the university clinics. Interviewers asked each survey question and then entered the participant's answers directly into a laptop-computer database; study staff then rechecked, verified, and exported data to permanent SAS (SAS Institute Inc, Cary, NC) files for cleaning, reporting, and analysis.

### Statistical analysis

We computed sampling fractions for each diagnosis–sex–ethnicity stratum as the ratio of the number of participants that completed an interview to the total number of clinic patients in the stratum. Differences between Hispanic and NHW populations were determined by Pearson chi-square test or Wilcoxon test. Each observation was weighted by the inverse of the appropriate sampling fraction, the Horvitz–Thompson weight, to obtain estimates of proportions, means, and odds ratios (ORs) for the target clinic population. SUDAAN version 9.0 (Research Triangle Institute, Research Triangle Park, NC) was used for all analyses. A stratified sampling with replacement design was specified, and sampling weights were assigned as described previously. Unless otherwise stated, it should be assumed that any reported statistic, other than sample size, is weighted to the target population.

## Results

### Group characteristics and demographics 

Of the total clinic population with osteoarthritis, 51.6% were Hispanic, and 48.3% were NHW. The Hispanic population did not represent a homogenous group. Most individuals within this group were born in the United States (86.3%) and described themselves mainly as Spanish American (78.8%) or Mexican American (14.5%). Of those born in Mexico (11.2%), all described themselves as Mexican or Mexican American. Only 2.5% were born in a country other than the United States or Mexico. Of these, two individuals self-identified as Central American and four as Cuban American.

Table 1 shows participant demographics for the entire sample and stratified by ethnicity. The Hispanic clinic population with osteoarthritis had significantly lower levels of education (*P* < .001) and lower annual household income (*P* < .001) than NHWs. Hispanic participants reported significantly higher (*P* < .001 for each) pain levels in the week before the interview, more mobility limitations, and less confidence in their ability to manage their arthritis than NHWs.

We further described Hispanic participants by acculturation. Language acculturation scores ranged from 5 (speaking, reading and thinking in Spanish only) to 25 (using English only) with a median of 15 on Marin's 5-item language use scale (30). Hispanics were classified as low-acculturated if they scored at or below the median, and as high-acculturated if they scored above the median. The median was set as a cut-off point for this study. As shown in Table 2, half (51%) of the Hispanic participants with osteoarthritis scored as high-acculturated, meaning they used English more than Spanish in day-to-day life, and half (49%) scored as low-acculturated, meaning they used Spanish more than English. High-acculturated Hispanic osteoarthritis patients were more likely to be women, to be less than 55 years old, and to have completed high school. Low-acculturated Hispanic osteoarthritis patients were more likely to report higher pain (*P* = .004) and disability levels (*P* = .002) and to report less confidence in their ability to manage their arthritis (*P* = .005).

### Differences between Hispanics and NHWs in patterns of current CAM use 

The vast majority (89.4%) of the osteoarthritis clinic population had ever used CAM, and 66.7% showed current use of CAM (data not shown). We chose *current use* of CAM therapies, defined as use at the time of the participant interview, as a more meaningful marker than *ever used* in these analyses because ever used included therapies that were tried only once or twice and then abandoned. As shown in Table 3, similar rates of current CAM use were observed for the two ethnic groups (65.5% for Hispanics and 67.8% for NHWs). However, Hispanics and NHWs varied in the kinds of CAM used. NHWs were significantly more likely to use nutritional supplements (*P* < .001), especially glucosamine, chondroitin, and flaxseed oil, than Hispanics. Significantly more Hispanics used oral herbs (*P* = .03), specifically garlic, and *items worn*, such as copper jewelry and magnets (*P* = .03), than did NHWs.

### Differences between groups of Hispanics by level of acculturation 

We further differentiated patterns of CAM use among the Hispanic population by level of acculturation as measured by the 5-item language-use scale. As shown in Table 4, low-acculturated Hispanics (66.0%) and high-acculturated Hispanics (65.1%) had similar overall rates of current CAM use. However, low-acculturated Hispanics were significantly more likely to use oral herbs (*P* = .03) and topical herbal rubs (*P* = .048) and less likely to use movement therapies (*P* = .009) than high-acculturated Hispanics.

### Logistic regression models for CAM use

Logistic regression modeling was undertaken to remove the potentially confounding effects of age, sex, education, income, duration of disease, pain, disability, arthritis helplessness, and medical skepticism from the crude association of ethnicity and CAM use. Ethnicity was categorized as NHW, low-acculturated Hispanic, or high-acculturated Hispanic. Table 5 presents the results as ORs with 95% confidence intervals (CIs), comparing low-acculurated and high-acculturated Hispanics as well as each group of Hispanics with NHWs. Although the general pattern of differences was similar to the crude differences shown in Tables 3 and 4, the pattern of statistically significant effects was somewhat different. High-acculturated Hispanics were more similar to NHWs in their use of most types of CAM than they were to low-acculturated Hispanics. Compared with NHWs, low-acculturated Hispanics had significantly higher use of oral herbs (*P* = .006) and items worn (*P* = .01), although high-acculturated Hispanics did not have higher use of oral herbs (*P* = .44) or items worn (*P* = .93).

### Communication with providers about CAM use 

Table 6 shows that Hispanics (52.2%) were significantly less likely than NHWs (75.2%) to report their CAM use to their primary care provider (*P* < .001). We asked the participants open-ended questions about why they told or did not tell their provider about CAM use and how the provider reacted. We then grouped, labeled, and coded responses for quantitative frequency counts. Only responses given by at least 6% of one of the two ethnic groups are shown in Table 6. In both ethnic groups, the key to CAM use disclosure or nondisclosure was whether or not providers directly asked about CAM use. NHWs were more likely to report a supportive reaction from their provider than Hispanics (*P* = .007).

### Other results 

Both ethnic groups had similar reasons for using CAM, although NHWs (5.0%) were more likely than Hispanics (0.4%) to say they preferred CAM to use of standard Western medications (*P* = .01). NHWs (7.8%) were also more likely than Hispanics (2.6%) to say that CAM improved their mobility (*P* = .04) (data not shown). For both Hispanics and NHWs, the primary reasons cited for using CAM related to pain relief; both groups were also interested in preventing disease progression or were willing to try CAM therapies to see if they were effective. CAM use did not change the use of standard medical therapies of most Hispanics (85.7%) or NHWs (85.4%) (data not shown). Of participants in both groups who reported a change in medical therapies, respondents primarily believed they would use fewer medications or be able to blend their CAM use with more standard medical therapies.

## Discussion

One of the most striking findings of this survey was the confirmation of high rates of overall use of CAM therapies for managing osteoarthritis among both Hispanics and NHWs with low- to mid-level incomes in a primary care setting. Most (89.4%) participants reported ever having used CAM, and 66.7% reported current use of CAM for osteoarthritis management, even after excluding high-response items, such as prayer, from the analyses. These rates are higher than most that have been reported in the literature. Barnes et al showed current rates of CAM use (for any purpose, including prayer) close to 62%, with lifetime use rates of 75% (3). Other surveys of CAM use have reported rates of current CAM use from 21% to 43% (4,7,9,33); higher rates of current use (34% to 66%) have been found in surveys of individuals with arthritis (8,13,14,17-20,34). New Mexico has a reputation for being alternative-health care friendly, which may help explain the numbers reported in this study. There are many alternative medicine practitioners in the Albuquerque area, and many people of different backgrounds and health care philosophies reside in this region. The environment also may contribute to the normalization of CAM practices and subsequent openness in discussing use of CAM therapies with primary care providers. Finally, the personal nature of the one-on-one interviews in this study may also have contributed to a more comfortable atmosphere and thus more honest revelations of CAM use.

Both Hispanics and NHWs in this population had similar rates of overall CAM use. Previous research has shown a range of 41% to 58% in overall rates of current CAM use among Hispanics (33,35-37). The high rates of use among Hispanics in this study can be explained in part by recognizing that our research focused on individuals with arthritis, who tend to report higher rates of CAM use overall. In contrast to our data, recent studies have shown higher rates of CAM use among Hispanics than among other ethnic groups, leading investigators to conclude that folk remedies or traditional healing practices are used more often in the Hispanic community (35,36). The similarity in rates of use of any type of CAM that we observed between the two ethnic groups may reflect higher than usual rates of CAM use among the NHW population.

Despite similar overall rates of CAM use, patterns of CAM use varied by ethnicity. After modeling to account for ethnic differences in demographics and disease status, we found that more low-acculturated Hispanics used oral herbs and wore items to manage their osteoarthritis than NHWs and that more high-acculturated Hispanics used energy therapies than NHWs. Previous research found that Hispanics were more likely to use traditional healing methods, often in the form of herbal preparations (33,35,36). It would make sense that degree of proximity to a parent culture would affect the degree of parent cultural influence on health care practices. This idea is supported by our findings that patterns of CAM use among high-acculturated Hispanics largely mirrored patterns among NHWs. On the other end of the spectrum, low-acculturated Hispanics were more likely than NHWs to use herbs, magnets, and copper jewelry. Although we controlled for the influence of age, sex, income, education, and disease status on CAM use, we did not control for other aspects influencing CAM use such as access to CAM services and products. Higher rates of use of herbal preparations and items worn among Spanish-speakers may be supported by a tradition of self-medication practices common in Central America, easy access to CAM products across the border in Mexico, the traditionally lower cost of gathered herbs, and the high degree of influence of family members and oral tradition on Hispanic health care practices (4,11,35,36). However, rates of overall CAM use among Hispanics in this study were not influenced by level of acculturation. Few studies have examined the influence of acculturation on CAM use; in Najm's study of the ethnic elderly, however, recent immigration status was predictive of CAM use among Hispanics (35).

Finally, these data reinforce the idea that communication between patients and primary care providers about the use of complementary therapies depends largely on whether the providers directly ask about CAM use. Although most participants from both ethnic groups reported telling their primary care provider about their CAM use, significantly fewer Hispanics (52.2%) did so than NHWs (75.2%). Furthermore, although Hispanics were less likely to communicate their CAM use to their primary care provider, they were also less likely to view their provider's response as supportive. These findings are fairly consistent with previous research showing that up to 66% of Hispanics never discuss their CAM use with their primary care provider (37). However, communication is not a problem limited to the Hispanic population; 25% of NHWs in this study and up to 83% of participants in other studies did not reveal their CAM use to their primary care provider (2,35,38). To a large extent, this lack of communication may be attributed to a don't-ask–don't-tell attitude, as first suggested by Eisenberg in 1998 and supported in subsequent literature (2,38). It is possible that patient–provider communication practices about CAM use may have shifted in the 4 years since these data were collected.

This study is not without its weaknesses or limitations. The data were derived from personal recollections of CAM use and are thus subject to recall bias. Recall bias may affect the accuracy of memory for types and frequency of use of CAM modalities, but more importantly it may blur the distinction between CAM use for arthritis and CAM use for general health maintenance or treatment of concurrent ailments. We hope that a focus on *current use* of CAM therapies may have avoided some of this effect. Also, osteoarthritis diagnosis was not confirmed by medical record review, so it is possible the sample included some individuals with chronic joint pain but without true osteoarthritis. The question may be raised of whether the five-question language-use scale, selected for a balance of convenience and validity, represents a true measure of acculturation. A process as complex and multifactorial as the assimilation of new cultural practices into an older tradition would be difficult to condense into any survey, let alone a short scale that only assessed use of the Spanish and English languages.

Generalizability of the study is limited for several reasons. As stated above, Albuquerque is a CAM-friendly area, so CAM use is likely to be higher in Albuquerque than in other regions. The information presented here was collected from a clinic-based population recruited from a single health care organization and may not be representative of other clinic populations. We do not know language acculturation levels in the regional Hispanic population and so cannot compare the acculturation results to a larger Hispanic population. We had low response rates overall, with a moderate difference in response rates between Hispanics and NHWs. However, nonparticipants were statistically similar to participants in sex and age. Finally, as is true of any research conducted regionally, findings gleaned from this Hispanic population are not necessarily applicable to Hispanics elsewhere because of the wide heterogeneity of Hispanic populations in this country. Even if generalizability were compromised, the conclusion stands that patterns of CAM use for osteoarthritis management may vary among subgroups in ways that cannot be assumed, and therefore it is important for providers to directly ask about CAM use, regardless of the patient's background.

These data analyses reinforce the understanding that individuals of different ethnic and socioeconomic backgrounds are using CAM at high rates. Although in the current study, acculturation and ethnicity appear to influence the forms of CAM that might be used, they do not seem to influence overall rates of use. In other words, we cannot make assumptions about who is and is not using alternative therapies based solely on an individual's cultural background. We are now seeing more research into the safety and efficacy of various CAM modalities. This research is allowing us to formulate increasingly clear-cut recommendations and treatment guidelines for CAM modalities. However, even if we are not able to provide our patients with specific recommendations, we must continue to monitor which therapies they are using on their own. It is clear that individuals are still not likely to disclose their use of CAM therapies in the absence of a direct question, and Hispanics may be more reluctant than NHWs to discuss their CAM use. In the end, it is the ethical obligation of the health care provider to ask patients about their use of CAM not only to facilitate an open patient–provider relationship but also to avoid possible drug–herb interactions and to provide guidance. Finally, as suggested in the 2004 summary on patterns of CAM use in the United States (6), we should continue to investigate where people are learning about CAM and take steps to improve the completeness and reliability of available information about CAM therapies.

## Figures and Tables

**Table 1:** Characteristics of Primary Care Patients With Osteoarthritis in University-based Outpatient Clinics in Albuquerque, NM, 2001–2002, Percentages Weighted by Inverse of Sampling Fraction

**Characteristic**	** Total Sample (n = 422)**	**Hispanic (n = 218)**	**Non-Hispanic White (n = 204)**	***P* Value^a^**
**Sex, %**				
Female	66.6	65.6	67.5	.46
Male	33.4	34.4	32.5	
**Age, %**				
18-54 y	21.5	20.8	22.1	.47
55-64 y	34.9	37.9	32.1	
65-74 y	27.9	28.2	27.6	
75-84 y	15.7	13.0	18.2	
**Education, %**				
Did not graduate from high school	28.9	53.0	6.7	<.001
High school graduate or graduate equivalency degree	19.5	23.5	15.9	
Some college	21.9	12.3	30.7	
College graduate	29.5	10.7	46.7	
Unknown	0.2	0.4	0	
**Annual household income, %**				
<$25,000	64.4	83.9	46.6	<.001
$25,000–$50,000	18.2	5.4	29.9	
>$50,000	12.8	4.0	20.8	
Refused or unknown	4.6	6.7	2.7	
**Duration of disease, y, weighted mean**	12.6	11.3	13.7	.04
**Pain index^b^, weighted mean**	5.3	6.1	4.7	<.001
**Disability index^c^, weighted mean**	1.0	1.2	0.9	<.001
**Arthritis Helplessness Index^d^, weighted mean**	13.2	14.9	11.9	<.001
**Medical skepticism scale^e^, weighted mean**	12.5	12.4	12.6	.66

a *P* values were determined by Pearson chi-square test or Wilcoxon test.

b The Wong-Baker Faces Pain Scale (27) was used to assess pain on a scale of 0 to 10, with 10 indicating greatest pain.

c The Stanford Health Assessment Questionnaire (24) was used to measure functional ability on a scale of 0 to 3, with 3 indicating greatest disability.

d The Arthritis Helplessness Index (25) was used to measure perceived ability to manage one's arthritis on a scale of 5 to 25, with 25 representing greatest helplessness.

e Fiscella's medical skepticism scale (26) was used to measure attitudes toward conventional medical therapy on a scale of 4 to 20, with 20 representing greatest skepticism.

**Table 2:** Characteristics of Low-Acculturated^a^ and High-Acculturated Hispanics Among Hispanic Outpatient Clinic Patients With Osteoarthritis in Albuquerque, NM, 2001–2002, Percentages Weighted by Inverse of Sampling Fraction

** Characteristic**	**Low Acculturation (n = 104)**	**High Acculturation (n = 106)**	***P* Value^b^**
**Sex, %**			
Female	58.4	72.6	.03
Male	41.6	27.4	
**Age, %**			
18-54 y	12.1	29.8	.01
55-64 y	39.6	38.9	
65-74 y	31.2	21.2	
75-84 y	17.1	10.1	
**Education, %**			
Did not graduate from high school	69.4	36.5	
High school graduate or graduate equivalency degree	16.3	31.3	<.001
Some college	6.8	17.6	
College graduate	6.6	14.5	
Unknown	0.8	0.0	
**Annual household income, %**			
<$10,000	42.8	40.8	.15
$10,000-$15,000	28.9	23.1	
$15,000-$25,000	11.5	21.1	
≥$25,000	7.8	11.6	
Refused or unknown	8.9	3.4	
**Duration of disease, y, weighted mean**	11.2	11.2	.98
**Pain index^c^, weighted mean**	6.6	5.6	.004
**Disability index^d^, weighted mean**	1.4	1.1	.002
**Arthritis helplessness index^e^, weighted mean**	16.0	14.1	.005
**Medical skepticism scale^f^, weighted mean**	12.7	12.2	.25

a Acculturation status was determined using a five-item scale that measured the extent of use of Spanish, English, or both languages in day-to-day life. Acculturation scores ranged from 5 (speaking, reading, and thinking in Spanish only) to 25 (speaking, reading, and thinking in English only) with a median of 15. Hispanics were classified as *low acculturated* if they scored at or below the median, and as *high acculturated* if they scored above the median.

b *P* values determined by Pearson chi-square test or Wilcoxon test.

c The Wong-Baker Faces Pain Scale (27) was used to assess pain on a scale of 0 to 10, with 10 indicating greatest pain.

d The Stanford Health Assessment Questionnaire (24) was used to measure functional ability on a scale of 0 to 3, with 3 indicating greatest disability.

e The Arthritis Helplessness Index (25) was used to measure perceived ability to manage one's arthritis on a scale of 5 to 25, with 25 representing greatest helplessness.

f Fiscella's medical skepticism scale (26) was used to measure attitudes toward conventional medical therapy on a scale of 4 to 20, with 20 representing greatest skepticism.

**Table 3:** Estimates of Current Use of Complementary and Alternative Medicine (CAM) by Ethnic Group Among Primary Care Patients with Osteoarthritis, Albuquerque, NM, 2001–2002, Percentages Weighted by Inverse of Sampling Fraction

**CAM Therapy^a^**	**Hispanic, Weighted %**	**Non-Hispanic White, Weighted %**	**Pearson Chi-Square *P* Value**
**Any CAM**	65.5	67.8	.63
**Nutritional supplements**			
Any type	25.3	42.4	<.001
Glucosamine	15.4	34.1	<.001
Chondroitin	11.2	24.0	.001
MSM (methylsulfonylmethane)	3.6	5.5	.33
Flaxseed oil	0.0	3.6	.007
Vinegar	6.4	2.8	.10
Fish oil	1.8	4.2	.17
**Vitamins and minerals**			
Any type	12.4	11.8	.85
Vitamin C	6.5	2.0	.03
Vitamin E	4.4	4.2	.91
Magnesium	2.9	3.9	.50
Vitamin B12	2.5	3.7	.60
**Oral Herbs**			
Any type	14.0	6.6	.03
Garlic	7.6	1.5	.005
**Topical herbal rubs**			
Any type	26.7	19.8	.10
Tiger balm	5.0	5.2	.95
Volcanico	5.0	0.4	.005
Capsaicin cream	4.3	4.8	.82
**Items worn**			
Any type	11.5	5.4	.03
Magnets	6.2	3.0	.13
Copper jewelry	5.7	2.8	.16
**Mind-body therapies**			
Any type	20.0	27.1	.12
Relaxation techniques	6.3	13.4	.03
Meditation	8.1	12.0	.22
Breathing techniques	5.8	10.7	.09
Sing or play instrument	7.7	4.9	.26
Visualization	6.1	7.4	.64
**Energy therapies**			
Any type	9.1	6.2	.30
Acupressure	3.9	1.1	.09
**Movement therapies**			
Any type	6.1	10.6	.13
Yoga	1.6	7.9	.008
**CAM therapists**			
Any type	6.9	8.4	.57
Massage therapists	3.5	6.0	.24
**Dietary approaches**			
Any type	4.8	7.9	.24

a Table shows only modalities used by at least 3% of one of the two ethnic groups.

**Table 4:** Estimates of Current Use of Complementary and Alternative Medicine (CAM) by Acculturation Status^a^ Among Hispanic Primary Care Patients With Osteoarthritis, Albuquerque, NM, 2001–2002, Percentages Weighted by Inverse of Sampling Fraction

**Category of CAM**	**Low Acculturation, %**	**High Acculturation, %**	**Pearson Chi-Square *P* Value**
Any CAM	66.0	65.1	.89
Nutritional supplements	24.9	27.4	.67
Vitamins and minerals	14.7	9.5	.28
Oral herbs	20.1	9.1	.03
Topical herbal rubs	33.1	20.8	.048
Items worn	13.7	8.3	.24
Mind-body therapies	16.3	23.5	.20
Energy therapies	6.0	12.8	.11
Movement therapies	1.8	10.7	.009
CAM therapists	8.0	6.2	.62
Dietary approaches	7.0	3.0	.21

a Acculturation status was determined using a five-item scale that measured the extent of use of Spanish, English, or both languages in day-to-day life. Acculturation scores ranged from 5 (speaking, reading, and thinking in Spanish only) to 25 (speaking, reading, and thinking in English only) with a median of 15. Hispanics were classified as *low acculturated* if they scored at or below the median, and as *high acculturated* if they scored above the median.

**Table 5:** Logistic Regression^a^ of Acculturation^b^ on Odds of Current Use of Complementary and Alternative Medicine (CAM) Among Hispanic and Non-Hispanic White (NHW) Outpatient Clinic Patients With Osteoarthritis in Albuquerque, NM, 2001–2002

**Category of CAM **	**Low-Acculturated Hispanic Compared With High-Acculturated Hispanic^c^ OR (95% CI)**	**Low-Acculturated Hispanic Compared With Non-Hispanic White^c^ OR (95% CI)**	**High-Acculturated Hispanic Compared With Non-Hispanic White^c^ OR (95% CI)**
Any CAM	1.18 (0.59-2.36)	1.24 (0.60-2.58)	1.05 (0.57-1.95)
Nutritional supplements	0.82 (0.41-1.63)	0.52 (0.26-1.05)	0.63 (0.34-1.18)
Vitamins and minerals	1.73 (0.65-4.60)	1.64 (0.62-4.37)	0.95 (0.37-2.41)
Oral herbs	2.61 (0.88-7.70)	3.99 (1.48-10.7)	1.53 (0.52-4.47)
Herbal topical rubs	1.39 (0.66-2.96)	1.48 (0.68-3.22)	1.06 (0.48-2.33)
Items worn	4.95 (1.75-14.0)	4.68 (1.45-15.1)	0.95 (0.25-3.54)
Mind-body therapies	0.81 (0.34-1.93)	0.89 (0.37-2.18)	1.11 (0.53-2.32)
Energy therapies	0.69 (0.21-2.28)	3.37 (0.93-12.2)	4.89 (1.57-15.2)
Movement therapies	0.18 (0.03-0.98)	0.31 (0.06-1.65)	1.71 (0.58-5.07)
CAM therapists	1.63 (0.45-5.83)	1.72 (0.52-5.63)	1.06 (0.38-2.94)
Dietary approaches	3.83 (0.91-16.0)	1.36 (0.34-5.40)	0.35 (0.08-1.49)

OR indicates odds ratio; CI, confidence interval.

a Model covariates are age, sex, income, education, duration of disease, pain index (27), disability index (24), Arthritis Helplessness Index (25), and medical skepticism scale (26).

b Acculturation status was determined using a five-item scale that measured the extent of use of Spanish, English, or both languages in day-to-day life. Acculturation scores ranged from 5 (speaking, reading, and thinking in Spanish only) to 25 (speaking, reading, and thinking in English only) with a median of 15. Hispanics were classified as *low acculturated* if they scored at or below the median, and as *high acculturated* if they scored above the median.

c Reference group.

**Table 6:** Communication With Primary Care Provider About Use of Complementary and Alternative Medicine (CAM) for Osteoarthritis Among Primary Care Outpatients by Ethnic Group, Albuquerque, NM, 2001–2002, Percentages Weighted by Inverse of Sampling Fraction

**Communication^a^ **	**Total Sample, Weighted % (n = 422)**	**Hispanic, Weighted % (n = 218)**	**Non-Hispanic White, Weighted % (n = 204)**	**Pearson Chi-Square *P* Value**
**Patient told provider about CAM use**	64.2	52.2	75.2	<.001
**Provider reaction**				
Supportive provider reaction	38.5	27.7	45.5	.007
Passive approval	29.8	39.1	23.7	.02
Neutral or no reaction	16.1	13.0	18.1	.30
Disapproved	3.4	6.8	1.2	.06
Provider responded by recommending further conventional therapies	2.7	6.1	0.5	.01
**Reasons for telling provider about CAM use**				
Because provider asked about CAM use	22.7	26.0	20.5	.36
Important to inform provider	31.3	29.1	32.7	.58
Might affect treatment choices	16.1	15.4	16.6	.82
Get provider's opinion	10.6	14.1	8.4	.18
Prevent drug interactions	7.6	4.9	9.3	.22
**Reasons for *not* telling provider about CAM use**				
Provider did not ask	37.8	42.5	28.7	.21
Not important to tell provider	23.3	21.1	27.6	.52

a Table shows only open-ended responses given by at least 6% of one of the two ethnic groups.
